# Crosslinguistic Influence (CLI) of Lexical Breadth and Depth in the Vocabulary of Bilingual Kindergarten Children – A Bilingual Intervention Study

**DOI:** 10.3389/fpsyg.2021.671928

**Published:** 2021-09-30

**Authors:** Minna Lipner, Sharon Armon-Lotem, Joel Walters, Carmit Altman

**Affiliations:** ^1^Department of English Literature and Linguistics, Bar-Ilan University, Ramat Gan, Israel; ^2^Gonda Multidisciplinary Brain Research Center, Bar-Ilan University, Ramat Gan, Israel; ^3^School of Education, Bar-Ilan University, Ramat Gan, Israel

**Keywords:** crosslinguistic influence, lexical breadth, lexical depth, bilingualism, bilingual intervention, kindergarten children

## Abstract

**Introduction:** Research in recent years has explored the vocabulary size (lexical breadth) of bilingual children, but less is known about the richness of bilingual word knowledge (lexical depth), and about how knowledge of words in the two languages interact. This study explores how bilingual narrative intervention with vocabulary instruction in each language may modulate crosslinguistic influence (CLI) between the languages of bilingual kindergarten children, focusing on CLI of lexical knowledge, and which factors modulate performance.

**Methods:** Forty-one typically developing English-Hebrew bilingual children (*M* = 64.63 months) participated. A bilingual adaptation of Story Champs narrative intervention program (Spencer and Petersen, 2012) was used to deliver vocabulary instruction in separate blocks of home language (HL) and school language (SL) sessions. Different intervention words were targeted in each language, but the children were tested on all target words in both languages. Lexical knowledge was assessed with a definition task four times throughout the study: prior to intervention, after each intervention block, and 4–6 weeks later. Learner characteristics (chronological age, age of onset of bilingualism and length of exposure) and proficiency in each language (standardized tests, familiarity with the vocabulary introduced in the intervention at baseline) were examined as possible modulators of performance.

**Results:** Children showed growth in lexical breadth and depth in their HL/English after HL intervention and in lexical breadth in the SL/Hebrew following SL intervention, with CLI for semantic depth observed via a qualitative analysis, but not quantitatively. Better HL/English performance was correlated with later AoB (and shorter SL exposure) and higher HL language proficiency scores. Children with higher HL/English proficiency responded better to the SL/Hebrew intervention, gaining more than those with lower English proficiency. Children with SL/Hebrew vocabulary dominance at the outset of the study also gained more from the HL/English intervention. No correlations were found between learner characteristics and SL performance.

**Discussion:** The current study indicates that bilingual narrative intervention with vocabulary instruction may be efficacious for improving the lexical breadth and depth of bilingual kindergarten children. It suggests that CLI may enhance bilingual children’s language learning success, and points to the importance of strengthening both languages of bilingual children.

## Introduction

Crosslinguistic influence (CLI), also known as crosslinguistic transfer, refers to the impact that a person’s knowledge of a language has on knowledge or use of another language (Jarvis and Pavlenko, 2008; Serratrice, 2013), and it can be bidirectional. With regard to lexical CLI, there is evidence that the effects may be facilitative (e.g., Bilson et al., 2015), with words learned in one language providing conceptual and semantic bases upon which a translation equivalent may be learned. Bilingual children may transfer semantic information between languages (Foroodi-Nejad and Paradis, 2009; Goodrich et al., 2016) as they gradually learn translation equivalents (Pearson et al., 1999). Given the importance of CLI for language learning, this paper aims to gain a deeper understanding of its modulators.

Intervention studies have generally investigated the effects of intervention on lexical growth within one of a bilingual’s languages (usually the societal language) (e.g., Carlo et al., 2004; Pham et al., 2018), but only a few have examined the effects of bilingual intervention (e.g., Restrepo et al., 2013; Armon-Lotem et al., 2020). In addition, most studies of vocabulary intervention have investigated effects on breadth of vocabulary (number of entries in a person’s lexicon), without studying growth in lexical depth (the amount of information about a word). There is a critical need to understand both what is learned and how much is learned, given the importance of lexical depth for later reading comprehension (Bialystok, 2006; Ouellette, 2006; Proctor et al., 2012). In addition, the majority of research has been conducted with Spanish-English bilinguals in countries where English is the societal language. Few studies have been conducted in contexts where English is a high-status heritage language, i.e., where patterns of CLI could be different. Despite the high status of English in the present study, as a heritage language it may still be subject to attrition (Ardila et al., 2019). Moreover, even among bilinguals with typical language development, the two languages are usually not balanced (Pearson et al., 1993; Bernardini and Schlyter, 2004; Kupisch, 2008; Paradis, 2010; Hoff et al., 2012) and support for the home language is often limited. At the same time, the societal language is important, especially for academic success, and therefore also needs to be strengthened. Thus, intervention in preschool years targeting high level vocabulary in both languages may contribute to academic success and promote additive bilingualism, in which both languages may develop. Finally, studying children with typical language development provides the necessary baseline for future studies of children with atypical language development. Beyond the practical implications of this study, exploring the CLI of semantic knowledge as a result of intervention is expected to contribute to understanding the organization of the lexical-semantic networks in this population.

The present paper explores how bilingual vocabulary intervention in the home and school languages (HL and SL) of bilingual kindergarteners impacts lexical breadth and depth in the HL and SL and may modulate the extent and nature of crosslinguistic transfer of semantic depth in the two languages. The aim is to document the intervention effects on CLI and explore the factors that may modulate these effects. In this vein, we examine children’s learner characteristics such as bilingual experience (chronological age, age of onset of bilingualism) and language proficiency in each language as potential modulators of CLI.

### Lexical Breadth and Depth in the Vocabulary of Bilingual Kindergarten Children

In the bilingual lexicon, the specific words known in each language may be different (David and Wei, 2008; Bialystok et al., 2010). Some words may only be encountered at home, in one language, and others may only be used at school, in a different language (Hoff and Core, 2013). Exposure to input in two languages from different caregivers in different contexts may also contribute to the unique breadth of vocabulary found in bilinguals resulting from their exposure to a wide variety of input in the two languages. Hence, bilingual children might not have translation equivalents for every word in their lexicons, raising the question as to whether there is any transfer of lexical and semantic knowledge between the HL and SL. This question is discussed below in the section on CLI.

The bilingual lexicon can also be examined in terms of lexical breadth and depth (also known as semantic depth). Lexical breadth refers to the number of entries in one’s lexicon. Lexical depth refers to the knowledge about those entries, viz. phonological, syntactic, collocational, morphological, semantic, pragmatic, and other information (Ordóñez et al., 2002). This information is acquired over time, through multiple encounters with each word. The relatively rich body of knowledge about vocabulary size, or lexical breadth, stands in contrast to the dearth of literature on lexical depth in bilinguals. Yet some scholars have noted that measuring lexical breadth without taking depth into consideration is of limited value (Wesche and Paribakht, 1996). At the initial stages of learning a word, children with “fast mapped” knowledge (Carey, 1978) know few aspects of a word, and may respond correctly on a receptive vocabulary measure but lack the ability to produce the word in conversation or use this knowledge when reading (Hadley et al., 2016). Additional experience with individual words increases the depth of children’s lexical knowledge. An information-rich context such as reading a book with pictures may enable gaining such additional knowledge from a single encounter (Hadley et al., 2016). The intervention in the current study presented words in a rich narrative context, with games and activities designed to broaden the contexts in which the words are found and deepen the knowledge of their use.

Tasks intended to tap into lexical depth include definitions (e.g., Snow, 1990), word association tasks (Schoonen and Verhallen, 2008; Sheng et al., 2012; Hai Weiss, 2020), and interviews that include giving definitions and answering questions about words (Ordóñez et al., 2002). Several studies of vocabulary depth in bilingual populations have found that depth is correlated across the two languages (Ordóñez et al., 2002; Dam et al., 2020), suggesting possible transfer of this kind of information. A recent study by Pham et al. (2018) investigated vocabulary learning following an HL intervention in several groups of bilingual children (ages 6–8). The study focused on several measures of vocabulary learning (identifying words in pictures, producing features of words, producing superordinate labels, and communicating word meaning effectively), which correspond to breadth and depth measures used in other studies (e.g., Ordóñez et al., 2002). Targets were eight words (four nouns, three verbs and one adjective), some of which were cognates. They found that the children improved in their HL on several measures of vocabulary, but only children with high HL proficiency who spoke a related language were able to transfer information to their SL, improving in definitions and communicating word meaning. These findings suggest that lexical depth may transfer.

However, with the exception of the Pham et al. (2018) study, there is a paucity of research investigating lexical depth and transfer effects in young bilingual children, especially following intervention. This gap motivated the present study in an effort to better understand the bilingual lexicon and explore ways to develop this important knowledge. It may lead to a better understanding of how bilingual children develop depth in their languages and how their language experiences and individual characteristics may contribute to this growth.

### Lexical and Semantic Crosslinguistic Influence (CLI)

Several models provide the theoretical basis for the current study. First, the Revised Hierarchical Model (Kroll and Stewart, 1994) posits shared conceptual representations for words as well as lexical links between words across languages. Indeed, much research has shown that when bilinguals use either one of their languages, words in both languages become active in parallel (Kroll and Ma, 2018). Accordingly, it may be posited that when a word is acquired in one language, knowledge of that word in the other language may be activated, facilitating transfer of semantic information. Second, Cummins’ (1979, 1981) linguistic interdependence hypothesis and his further work (2000, 2008) maintain that bilinguals have a common set of cognitive processes and a single representational system underlying their two languages, such that instruction in the HL should transfer to the SL and vice versa. Furthermore, he posits that knowing concepts in one language may expedite vocabulary learning in the second language, because the conceptual knowledge helps the child understand the meaning of the new unknown word. MacWhinney’s Unified Competition Model (UCM; MacWhinney, 2005) maintains that when there is some kind of linguistic match between the HL and SL, language learners will attempt to transfer knowledge if it is close enough (even if it is not a complete match). Thus, positive transfer of lexical meaning in cases of words with the same or similar meanings is expected.

Work in past years has shown that words in a bilingual’s two languages are mentally connected to each other, either directly (word-to-word) or through links to semantic representations (Jarvis and Pavlenko, 2008; Poulin-Dubois et al., 2013; DeAnda et al., 2016), thus providing the basis for transfer of lexical information between the two lexicons. MacSwan and Rolstad (2005) have suggested that young sequential bilinguals in the early stages of acquiring their second language may use the conceptual and lexical knowledge from their HL to assist them in learning words in SL. Indeed, Singh (2014) found crosslinguistic semantic priming effects from the dominant language to the non-dominant language in bilingual toddlers, indicating the existence of lexical stores that are conceptually related crosslinguistically.

A number of studies of bilinguals have shown that transfer of lexical knowledge occurs across languages and word learning in bilingual children is expedited by the second language (Ordóñez et al., 2002; Bilson et al., 2015; Goodrich et al., 2016; Armon-Lotem et al., 2020). However, most of these studies have focused exclusively on vocabulary breadth, without examining depth, leading to an incomplete understanding of lexical transfer.

#### Modulators of CLI

In addition to documenting the presence of CLI, it is important to understand the circumstances in which it occurs. Research on the modulators of CLI has shed some light on this issue. One area of interest in research on CLI modulators is language dominance and proficiency. Bilingual children often differ in the language proficiency of their two languages, and in fact, most bilinguals are dominant in one or the other language (Pearson et al., 1993; Bernardini and Schlyter, 2004; Kupisch, 2008; Paradis, 2010; Hoff et al., 2012), which could potentially affect the extent and direction of CLI. According to the Revised Hierarchical Model (Kroll and Stewart, 1994; Kroll and Ma, 2018), the degree of bilingual fluency modulates the link between the lexicons and the shared conceptual representations of words, with more proficiency implying stronger lexical and conceptual links between concepts and words across languages, which will lead to enhanced performance on language tasks in the dominant language. Thus, according to this model, language dominance should play a role in determining cross-language performance, as vocabulary development in the SL should be expedited when concepts are already known in the HL.

However, despite the theoretical basis and evidence for shared semantic representations in the bilingual lexicon, studies on vocabulary often focus on the factors leading to varying outcomes without relating to CLI (e.g., Paradis, 2011). One exception is a study by Altman et al. (2018), which examined the connection between vocabulary in one language and fast mapping in the other. In this study of Russian-Hebrew bilingual kindergarteners (ages 5–6), the authors found that the Russian (HL) dominant children used receptive vocabulary in Russian to support fast mapping in SL/Hebrew. A study by Kan and Kohnert (2012) also found crosslinguistic relations between HL vocabulary and word learning ability in SL in young sequential bilinguals (ages 3–5), with larger HL vocabularies supporting SL learning. However, the study did not investigate the factors affecting variation in CLI. The authors speculated that the children’s home environments may have accounted for the differences.

Another factor impacting CLI is age of onset of bilingualism (AoB), which is often related to language proficiency and dominance. Meir et al. (2017) found evidence for bidirectional CLI in the morphosyntax of 5–6 year-old children, showing that participants with earlier AoB to SL (Hebrew) (0–23 months) mastered the contrastive structures (articles, perfect aspect, case inflections) better in SL and performed worse in HL (Russian), while those with later AoB had better performance in HL and weaker acquisition of the same structures in SL. Thus, AoB affected the degree of CLI, with more impact of the HL occurring with later AoB for structures that differed in the two languages. Likewise, Chondrogianni and Marinis (2011) evaluated the influence of AoB, length of exposure, and other factors on children’s acquisition of SL vocabulary (and morphosyntax) and found that age of onset and length of exposure were significant predictors of vocabulary level: later AoB and longer exposure led to enhanced performance, suggesting that increased exposure may facilitate vocabulary development. In contrast, Unsworth (2016) did not find effects of AoB in the SL acquisition of vocabulary when she compared bilinguals with AoB of 1–3 years versus 4–7 years. These mixed findings for AoB could be related to the relation between quantity of input, vocabulary knowledge, and transfer effects (Pearson et al., 1997; Vermeer, 2001; Poulin-Dubois et al., 2013), which is beyond the scope of the present study.

Some intervention studies have focused on the language of instruction as a variable in performance (Ebert et al., 2014). Lugo-Neris et al. (2015) investigated the order of the language of intervention for a group of Spanish-English bilinguals at risk for language impairment, and found that those who received initial intervention in HL/Spanish (the more proficient language for most of the children) made larger gains than those who began in SL/English. This finding provided the motivation in the current study for intervening first in the HL/English, as it was the more dominant language for most of the children. To sum up, research on factors modulating CLI has not produced a clear picture of the conditions that facilitate crosslinguistic transfer of lexical knowledge.

### Vocabulary Intervention Studies

In addition to the intervention studies mentioned above, which focused on language of instruction as a variable, a number of other vocabulary intervention studies have been conducted among bilingual children. Carlo et al. (2004) conducted a vocabulary intervention with monolingual and bilingual fifth graders, providing the bilingual children with materials in their native language (Spanish) to preview before the English language intervention. They found that all children in the intervention group improved on vocabulary measures including measures of vocabulary depth, as well as on comprehension, with gains for bilinguals similar to those of the monolingual students. Investigating the role of the language of vocabulary instruction with preschool Spanish-English children, Mendez et al. (2015) compared the effects of instruction in English only versus bilingual instruction using various instructional strategies. They found that immediately following instruction, the children in the bilingual instructional condition knew significantly more vocabulary words in SL than the children in the English-only group. The study also reported, as did previous research (Rolstad et al., 2005; Restrepo et al., 2013), that children exposed to bilingual instruction gained vocabulary in HL as well. The authors speculated that use of HL for presenting words before introducing them in SL allowed children to use their existing linguistic and conceptual HL knowledge to facilitate SL acquisition.

However, these studies introduced the same words in both languages, thereby drawing explicit connections between the words. The question remains as to whether words and concepts introduced in one language only will be connected to information about the words in the other language without explicit connections being made between them. That is, if a concept is introduced in one language, according to the Revised Hierarchical Model (Kroll and Stewart, 1994), the lexical knowledge of that concept should be activated (Kroll and Ma, 2018), allowing for transfer of the new knowledge. The current study allows for exploration of this hypothesis.

A recent study by Armon-Lotem et al. (2020) provides some evidence to support this assumption. Sixteen English-Hebrew bilingual preschoolers underwent a bilingual narrative intervention (BINARI) with vocabulary instruction, using a design similar to the one reported in the present study. The children made progress in vocabulary breadth with gains in the language of intervention as expected. Cross-linguistic gains were observed in SL/Hebrew following the HL/English intervention, but no gains were observed in HL/English following the SL/Hebrew intervention. This study was conducted with a small group of children with no control group and did not distinguish between words taught in the intervention and translation equivalents. Further work is needed to determine whether and under what circumstances information may transfer in both directions as a way of better understanding the nature of vocabulary depth in bilingual children. The Pham et al. (2018) study reviewed above provides some evidence of transfer of lexical information, including depth, but it was conducted with a small number of words, half of which were cognates.

Taken together, these studies demonstrate that vocabulary interventions can be successfully implemented with bilingual populations. Intervention studies focusing primarily on vocabulary indicate that bilingual intervention has positive effects on both languages, whereas intervention only in SL impacts the SL only and may have detrimental effects on HL (Restrepo et al., 2013). Armon-Lotem et al. (2020) found that all children showed gains in SL following intervention in HL, but only children who were relatively stronger in HL gained in HL following SL intervention. This result is one of the motivations for intervening in and testing both languages. The impact of intervention in each language separately and the cumulative effect of intervention in two languages will be addressed to better understand the nature of transfer of lexical knowledge across languages.

### The Present Study

The present study investigated the effects of a bilingual narrative intervention with embedded vocabulary instruction on the lexical breadth and depth of English-Hebrew bilingual kindergarten children. Administering intervention in both languages for different words enabled an examination of CLI from one language to the other, as all the words were tested in both languages. Progress was measured with a definitions task, which was scored to measure growth in both vocabulary breadth and depth. The following questions are addressed:

1. Does bilingual narrative intervention (BINARI), in particular, vocabulary instruction, improve lexical breadth and depth in the HL and SL of English-Hebrew preschool children?

2. Do the learner characteristics of language dominance, higher proficiency, and AoB predict CLI of lexical breadth and depth?

3. How is CLI from the HL to the SL and from the SL to the HL manifested in lexical breadth and depth?

BINARI is anticipated to enhance lexical knowledge in children in both languages by increasing the breadth and depth of their vocabulary. CLI is expected to be greater for transfer of lexical information (depth) for familiar concepts, since increasing depth for an already known lexical form may occur more readily than for a novel form, as seen in previous research (Pham et al., 2018). By contrast, increasing lexical breadth with new concepts introduced in one language might take longer to impact the other language and lead to acquiring a word in the that language without further support. Finally, language dominance, higher proficiency and AoB (as a correlate of length of exposure) are predicted to facilitate CLI.

## Materials and Methods

### Participants

Forty-one typically developing (TLD) English-Hebrew bilingual preschool children aged 5–6 years old (*M* = 64.63 months) participated in the study. The children were recruited from preschools located in areas with a concentration of English-Hebrew bilingual families. Consent forms were sent to the parents of approximately 200 children in six kindergartens. Forty-three parents gave consent for their children to participate in the study and filled in a parental questionnaire about the children’s demographic and linguistic background. Two children were excluded due to very low performance on both the English and Hebrew screening test, which might indicate atypical language development (Håkansson et al., 2003). The remaining forty-one children were randomly assigned to two groups, one experimental, whose members received BINARI, the other, a control group. Criteria for inclusion in the study were: (1) one of the parents had to be a native English speaker; (2) the child had to score at or above local bilingual standards (see below) on either the English or Hebrew standardized language screening test (Armon-Lotem and Meir, 2016; Armon-Lotem et al., 2021); (3) the child had to score above 85 on the Raven Progressive Matrices non-verbal intelligence test (Raven et al., 1998); (4) the child had to be exposed to the L2 for at least 24 months; and (5) the child did not have any history of a hearing impairment or parental concerns about language. Written informed consent was obtained from the parents, who also provided information about their child’s language development and background. Children expressed oral assent to participate in the study and were allowed to terminate participation at any time. The study was approved by the Bar-Ilan University IRB and the office of the Chief Scientist of the Israeli Ministry of Education.

In order to assess the language performance of bilingual children in their home language (HL/English), the Clinical Evaluation of Language Fundamentals (CELF Preschool-2; Wiig et al., 2004) was used. The CELF-Preschool-2 (Wiig et al., 2004) consists of six subtests for concepts and following directions, word structure, expressive vocabulary, recalling sentences, sentence structure, and receptive and expressive word classes. Local bilingual standards for the Core Language Scores (CLS) of the CELF-Preschool-2 (with a cutoff point of -1.25 *SD*) are available for English-Hebrew bilinguals (Rose, 2018; Armon-Lotem et al., 2021). Data from 240 typically developing English-Hebrew bilingual children aged 5;0–6;5 years have been used to set the local standards for the CLS taking into account chronological age and age of onset of bilingualism, identifying the means and SDs which indicate typical development in English as a HL in Israel. To assess the children’s language performance in the school language (SL)/Hebrew, the *Goralnik Screening Test for Hebrew* (Goralnik, 1995) was administered. The test includes six subtests: sentence repetition, comprehension, expression, pronunciation, vocabulary, and storytelling. The scoring totals 180 points, 30 for each subtest. Local bilingual standards for the Goralnik raw score were used with a cutoff point of -1.25 SD (Iluz-Cohen and Armon-Lotem, 2013; Armon-Lotem, 2014; Altman et al., 2016). Data from 443 bilingual children aged 5;0–6;5 years speaking Hebrew as SL have been used to set the local standards for the Goralnik, taking into account chronological age and age of onset of bilingualism, by identifying the means and SDs which indicate typical development for SL/Hebrew in Israel.

Table 1 displays the children’s mean ages at the onset of the study, their age of onset of bilingualism (AoB), i.e., when they were first exposed to SL/Hebrew, years of mothers’ education as a proxy for socioeconomic status, and their results on the standardized language tests (mean Core Language Scores [CLS] for the CELF on a standard scale where the mean is 100 and SD is 15, and the mean raw scores for the Goralnik). The information from the standardized testing is used to compare the performance of the experimental group and the control group, not to compare the two languages within each group, as the two measures are not comparable. The standardized tests make it possible to compare the children’s performance in each language within the group to assess the impact of proficiency level on benefits from the intervention. No significant differences were found between the experimental and control groups in age and years of mothers’ education. The age of onset of bilingualism (AoB) differed significantly between the two groups, with the experimental group having a lower AoB than the controls. However, there was no significant difference between the groups in their language proficiency in either language.

**TABLE 1:** Background information for participants.

**Group**	**Mean age (in months)**	**Mean AoB (in months)**	**Mean years of mothers’ education**	**Raven standard score**	**HL/English proficiency: CELF–CLS**	**SL/Hebrew proficiency Goralnik raw scores**
Experimental (*n* = 22)	64.82 (3.74) (58–72)	17.18 (15.64) (0–38)	16.86 (2.44) (12–21)	116.89 (12.96) (90–132)	97.77 (11.96) (81–114)	129.14 (17.65) (90–157)
Control (*n* = 19)	64.42 (5.34) (57–75)	26.84 (10.86) (4–40)	16.05 (2.55) (12–21)	120.46 (11.70) (100–138)	99.74 (10.78) (81–125)	125.58 (13.14) (106–143)
Totals (*n* = 41)	64.63 (4.49) (57–75)	21.66 (14.33) (0–40)	16.49 (2.49) (12–21)	118.34 (12.40) (90–138)	96.68 (11.33) (79–125)	127.49 (15.63) (90–157)

*HL, home language/English; SL, school language/Hebrew; AoB, age of onset of bilingualism; HL assessed via CELF (Wiig et al., 2004); SL proficiency assessed via Goralnik (1995). Raven standard score is based on a subset of 17 children in the experimental group and 13 in the control group as no norms were found for the younger children.*

### Materials and Procedure

#### Overview

The BINARI intervention utilized in the study was based on *Puente de Cuentos* (Spencer et al., 2017), a Spanish-English bilingual adaptation of the *Story Champs* program (Spencer and Petersen, 2012), designed to enhance the narrative and vocabulary skills of Spanish-English bilingual preschool children (for details see Petersen and Spencer, 2016). Several adaptations were made to the program for use in the present study. First, the most culturally appropriate stories were selected, translated and adapted linguistically and culturally to the Israeli context. Second, ten stories were translated into Hebrew [six for the intervention sessions, and four for progress monitoring (PM)]. Third, the original five-picture format of the stories was expanded to include six pictures in order to better conform to the story grammar literature. Finally, following the results of a pilot study with 16 children (Armon-Lotem et al., 2020), six of the twenty-four vocabulary items were changed to better reflect the culture and level of this population.

The target vocabulary words were chosen based on the original *Puente de Cuentos* intervention, with adaptations made based on the pilot study. Six verbs and six adjectives were selected for intervention in each language to cover a range of difficulty (based on the pilot study and in consultation with preschool teachers). For the Hebrew adaptation, bilingualism experts and experienced preschool teachers were consulted to choose words that would be of an appropriate level in the absence of Hebrew frequency lists. The intent was to leave room for improvement on the one hand, but to make sure the task was not too frustrating on the other. This variation in difficulty also allowed for the diversity in the children’s abilities. In general, verbs and adjectives are considered more challenging for this age group (Johnson and Anglin, 1995; Blackwell, 2005) and are thus considered appropriate targets for language enrichment.

##### Design

**FIGURE 1:**
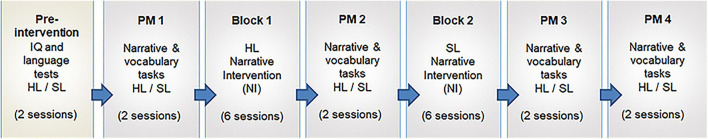
Protocol for data collection.

The intervention consisted of two blocks of six group sessions each: the first block was conducted in the HL (English), and the second in the SL (Hebrew). The children were evaluated four times in progress monitoring (PM) sessions: before the intervention, after each block of intervention, and 4–6 weeks after the end of the intervention, to check for maintenance. PM sessions took place in the children’s preschools or online via Zoom© in their homes (see “Covid-19 adaptations” for details below). The sessions were audio recorded. All research assistants were native speakers of the target languages (two for each language) and had extensive experience working with children this age. Figure 1 summarizes the protocol for data collection. The control group followed the same PM sessions protocol as the experimental group and at similar intervals, but did not undergo any intervention.

The majority (95%) of the intervention sessions took place in the children’s preschools, one to three times a week, depending on the preschool schedule, and totaled six sessions over 2–3 weeks in each language. Each session lasted 20–25 min.

##### Procedure

Intervention was conducted in groups consisting of three-four children. Each session began with a short introduction about the purpose of the session and a very brief review of the words learned in the previous session in that language. Then a new story was introduced, and the accompanying activities from the *Puente de Cuentos* manual (Spencer et al., 2017) were carried out as well as a supplementary vocabulary activity for each word at the end of the session. The targeted vocabulary words were first introduced in the context of a story, and then practiced individually, first with reference to the story content, and then in other contexts, thereby enriching the children’s knowledge of the words. Each session included multiple repetitions of the story, with telling and retelling by the experimenter and children. Thus, the words were introduced in a context rich in information and visual stimuli (Hadley et al., 2016) designed to develop vocabulary depth as well as enhance breadth. See Supplementary Appendix A for a sample intervention protocol.

#### Vocabulary Knowledge Task

The PM sessions included a Vocabulary Knowledge Task (designed for the present study and used in the pilot) to test the children’s knowledge of the target words and their translation equivalents. The vocabulary task was developed to assess the semantic content of the children’s definitions (Korat et al., 2014), as opposed to definitional form (Benelli et al., 2006).

The task was designed to parallel the intervention. In the intervention, the same 24 target words were taught to all participants, 12 in the English intervention and 12 in the Hebrew intervention. All 24 words were tested in the vocabulary task in each language: 12 English target words and 12 English translation equivalents for the Hebrew target words were used in English testing and 12 Hebrew target words and 12 Hebrew translation equivalents of the English target words were used in Hebrew testing. This procedure was designed to allow assessment of language transfer effects in both directions. The only difference between the Hebrew and English tasks were the stimulus sentences, which contained different content to ensure that the children would focus on the target words and not on the sentence contexts. Supplementary Appendix B contains the Vocabulary Knowledge Task for English and Hebrew.

In the task children were asked to define the target words and the translation equivalents (verbs and adjectives) introduced in the BINARI sessions. Generally, a child’s ability to explicitly formulate a qualitative definition is considered a clear indication that the word is known (Johnson and Anglin, 1995). Definition tasks have been used to measure depth of vocabulary knowledge (e.g., Read, 1993; Verhallen and Schoonen, 1993; Ordóñez et al., 2002). In the task, the words were read aloud to the children in short sentences designed to provide minimal contextual clues. The experimenter began by saying, “We’re going to play a game with words and sentences. I’m going to read you sentences with words. I want you to tell me what the words mean – whichever ones you know.” Children practiced on a sample word and sentence before starting the task. After each sentence, the experimenter asked what the target word meant. For example, for the target word “scrubbed,” the prompt was: “The boy *scrubbed* the floor. What does *scrubbed* mean?” Experimenters did not comment on responses. However, if the response was ambiguous, or if the child used a gesture instead of defining it verbally, the experimenter encouraged the child to elaborate by asking the child to explain the item verbally. The test was administered to all participants in the same order. Each language was tested on a different day by native speakers of that language. Responses to the vocabulary task were recorded manually during the sessions, with audio recordings used as backup.

#### Adaptations for Covid-19

Overall, 95% of the intervention was completed in face-to-face sessions. Following the onset of Covid-19, kindergartens were closed, so the final one or two sessions of the intervention were completed individually online for some of the children (*N* = 12). In addition, PM3 and PM4 were administered online for the experimental group, and PM2-4 were conducted online for the control group.

The online versions of the final intervention sessions and PMs were created to be as similar as possible to the face-to-face version and were discussed amongst experimenters for consistency and practiced before data collection. At the end of the first online session, each child was asked how he/she felt about doing it online. All children had experienced online learning from the beginning of the first lockdown, and most reported that they enjoyed it (some even said they liked it more than in the kindergarten). The four research assistants reported that the children cooperated very well online, and that they did not notice differences in their performance (other than the expected improvement following intervention).

### Data Scoring and Analysis

#### Coding and Scoring

Children’s responses were rated for their expressed knowledge of the words. The responses were coded as full definition, partial definition, codeswitching, gesture, wrong definition and “I don’t know.” A response was counted as a codeswitch when a codeswitched element was a meaningful and relevant part of the definition; all codeswitches consisted of single words. For example, when asked to define “heavy” in English, a child said “*kaved*” (heavy in Hebrew). Expressive lexical knowledge includes all answers that show knowledge of the word as well as codeswitching. Scoring was based on the content of the child’s response, not on the form of the definition. Scores for breadth were 0–1, reflecting no knowledge versus some/complete knowledge including codeswitching. Scores for depth were 0–1–2, where no response or an error received a score of zero (0), a partially correct answer (including associations and incomplete explanations) received one point (1), and complete knowledge received a score of two (2). A score of two was given only to responses that included a precise and/or complete definition and was perceived as an indicator of greater depth of knowledge. Less complete or precise responses that nevertheless showed some knowledge of a word received one point. Thus, a score of one on the breadth scale would receive a score of one or two on the depth scale. Codeswitches were scored as 0 for lexical depth as they were not indicative of expressive vocabulary in the tested language. Gestures were scored as 0 for both breadth and depth as they indicate non-verbal knowledge.

Tables 2A,B outline the coding and scoring for the lexical breadth (2A) and depth (2B) scales and give sample responses from the English vocabulary data. Ambiguous responses were discussed among the raters, and when necessary, a third researcher resolved the disagreement.

**TABLE 2:** Scoring for lexical breadth on the vocabulary knowledge task.

**A. Vocabulary Breadth Scale**		
**Scores**	**Types of answers**	Examples of answers*

0	- Wrong answer	- *SCRUB*: fall
	- Gesture	
	- I don’t know/no response	
1	- Correct/complete answer	- *COLLECT*: bring together
	- Correct answer using a form of the word to define the word (but indicating complete comprehension)	- *SCRUB*: to scrub the floor with a brush until it’s clean
	- Close/partially correct answer/incomplete explanation	- *SCRUB*: to clean (incomplete)
	- Association	- *COOPERATE*: to be good (association)
	- Correct codeswitch	

**B. Vocabulary Depth Scale**		

**Scores**	**Types of answers**	Examples of answers*

0	- Wrong answer	*DAMP*: really deep
	- Gesture	
	- Codeswitch	
	- I don’t know/no response	
1	- Correct answer using a form of the word for definition (but indicating comprehension)	- *SCRUB*: He scrubbed the stain with a sponge
	- Close/partially correct answer/incomplete explanation	- *SCRUB*: to clean (incomplete answer)
	- Association	- *TREMBLING*: you’re cold (association)
2	- Correct/complete answer	- *SCRUB*: to clean hard
		- *COOPERATE*: to work together
		- *TREMBLING*: shaking

*Target words are in all caps and italics.*

#### Analyses

The results are analyzed for PM 1-3. PM1 informs us of the relative performance of the children in each language at baseline, which makes it possible to identify dominance and the impact of the intervention. PM2 immediately followed the intervention in HL (English) enabling identification of gains in both languages and CLI in Hebrew following the English intervention. PM3 immediately followed the intervention in SL (Hebrew), and enables identification of gains in both languages and CLI in English following the Hebrew intervention. PM4 is reported only in the descriptive statistics of the full corpus (not in further analyses) since it tested for maintenance and does not contribute to the discussion of immediate gains and CLI.

Descriptive statistics are reported for overall distribution of responses (numbers and percentages out of total responses) reporting full responses, partial responses, codeswitching and gestures, followed by χ^2^ tests. Means and standard deviations calculated for the 0–1 scale were used in multivariate GLMs and one-way ANOVAs (with *post hoc* tests) to explore lexical breadth. Spearman correlational analyses and Linear Regressions were used to assess the impact of learner characteristics on gains to test for CLI. Crosstabs were used to compare the performance on the 0–1–2 scale to explore lexical depth. Manual analysis of the responses was used for the qualitative analysis to identify CLI in lexical depth.

To ensure procedural fidelity during the intervention sessions, a procedural reliability form consisting of all the steps in the intervention session was filled out. These records show that the procedure was implemented with over 99% reliability. To establish inter-rater reliability for the vocabulary task coding, 25% of the children’s responses in each language were double coded by the authors and two graduate students who conducted the intervention and coded the data. The agreement percentage was calculated by dividing the number of discrepant scores over the total number of answers. Reliability for the coding was initially 94% for English and 92% for Hebrew, but after discussion and adjustments, full agreement was reached among raters.

## Results

### Descriptive Statistics

The vocabulary knowledge task resulted in 4200 responses by the experimental group and 3528 responses by the control group. Table 3 presents the overall distribution of the responses (numbers and percentages out of total responses) divided by Group and Language of Testing. The number of PMs is reported for each language in each group (N = number of children multiplied by four excluding missing children). Due to Covid19, one child in the experimental group was absent for one Hebrew PM, one child in the control group missed one English PM and four children in the control group missed one PM each. Total responses are the number of responses provided by the children and include full responses (depth score of 2), partial response (depth score of 1), codeswitches and gestures as well as wrong definitions and “I don’t know”. The latter two categories are not presented in the table. Chi-squared (χ^2^) tests were applied to the different types presented in the table for within group and between group comparisons.

**TABLE 3:** Descriptive statistics of the full corpus of responses.

**Group**	**Experimental**		**Within χ^2^**	**Control**		**Within χ^2^**	**Between groups χ^2^**	
**Language of testing**	**English [*N* = 88]**	**Hebrew [*N* = 87]**		**English [*N* = 75]**	**Hebrew [*N* = 72]**		**English**	**Hebrew**
Total responses	2112	2088		1800	1728			
Full responses	546 (26%)	424 (20%)	18.18***	275 (15%)	274 (16%)	ns.	65.53***	209.56***
Partial responses	425 (20%)	185 (9%)	107.2***	462 (26%)	128 (7%)	76.07***	17.03***	ns.
Code-switching	1 (0%)	10 (0.5%)	7.486**	0 (0%)	28 (1.6%)	27.09***	ns.	12.49***
Gestures	22 (1%)	62 (3%)	19.9***	10 (0.5%)	30 (1.7%)	10.96***	ns.	6.11*

*ns.* = non-significant, *p < 0.05, **p < 0.01, and ***p <0.001.

Within group comparisons show English dominance for both groups. Within the experimental group, more full responses and more partial responses were provided in the English testing than in the Hebrew testing, while more codeswitches and gestures were used in the Hebrew testing. Within the control group, percentages of full responses were the same in English and Hebrew, but there were significantly more partial responses in English than in Hebrew. Like in the experimental group, more codeswitching and gestures were used in Hebrew than in English. Between group analyses demonstrate the impact of the intervention on both languages. The between group analysis shows that the experimental group gave more full responses in both languages and fewer partial responses in English, with no significant difference on partial responses in Hebrew. The experimental group used codeswitching less but gestures more in Hebrew when compared to the control group, with no difference in English for these measures.

In order to further explore the effect of bilingual narrative intervention (BINARI) that includes explicit vocabulary instruction on the lexical breadth and depth of the HL and SL lexicons of English-Hebrew preschool children, we then present the impact of the intervention on breadth and depth, comparing the experimental and control groups (Research Question 1). Then, focusing on immediate gains following the explicit vocabulary intervention, we explore whether learner characteristics such as bilingual experience and language proficiency predict performance and CLI for lexical breadth in the experimental group (Research Question 2). Finally, we focus on how cross-language influence from the HL to the SL and from the SL to the HL is manifested in lexical depth within the experimental group (Research Question 3).

### Impact of Lexical Intervention on Lexical Breadth in the HL and SL Lexicons

Data is reported for PM 1–3. PM4 for is not reported, as no significant changes were observed, showing maintenance of learning over time, but this does not contribute to the primary focus of the paper on immediate gains and CLI. Table 4 presents the ratio of items the children were able to verbally define (including full and partial definitions and codeswitching) out of the 24 items tested in each language at the progress monitoring points for the experimental and control groups. PM1 shows the relative performance of the children in each language at baseline, which makes it possible to identify the impact of the intervention. PM2 immediately followed the intervention in the HL (English). PM3 immediately followed the intervention in the SL (Hebrew).

**TABLE 4:** Mean scores for vocabulary breadth in each progress monitoring session in each language (reported as ratios).

**Group**	***N***	**Language**	**PM1**	**PM2**	**PM3**
Experimental	22	HL (English)	0.33 (0.20)	0.49 (0.22)	0.53 (0.19)
		SL (Hebrew)	0.24 (0.13)	0.26 (0.17)	0.33 (0.21)
Control	19	HL (English)	0.32 (0.14)	0.37 (0.14)	0.42 (0.15)
		SL (Hebrew)	0.20 (0.13)	0.25 (0.10)	0.24 (0.11)

*Language, language of testing; HL, home language; SL, school language; PM, progress monitoring.*

A multivariate GLM with Group (Experimental, Control), Language of Testing (HL/English, SL/Hebrew) and PM point (1–3) as independent variables and vocabulary knowledge as the dependent variable yielded significant main effects for Group, *F*(1,228) = 9.06, *p* = 0.003, η*^2^* = 0.04, Language of Testing, *F*(1,228) = 53.48, *p* < *0.001*, η*^2^* = 0.19, and PM point, *F*(2,228) = 8.21, *p* < *0.001*, η*^2^* = 0.07, with no interaction between these variables. The experimental group demonstrated better performance than the control group. Performance in English was better than in Hebrew, and the children progressed across PM points.

Since half of the target items were taught in Hebrew and the other half in English, and children were tested on all target items and their translation equivalents in both languages, a second analysis was conducted, comparing performance on target items taught in the language of testing and their translation equivalents (those not taught in the language of testing) at the three PM points. A multivariate GLM with Language of Intervention (HL/English, SL/Hebrew) as a repeated factor within participants, Group (Experimental, Control) as independent variables between participants, Language of Testing (HL/English, SL/Hebrew), and PM point (1–3) as independent variables between items and vocabulary knowledge as the dependent variable was conducted to test the effect of language of intervention on growth in vocabulary breadth. Results demonstrated a significant main effect for Language of Intervention, *F*(1,228) = 41.41, *p* < 0.001 η*^2^* = 0.15, and significant interactions between (a) Language of Intervention and Language of Testing *F*(1,228) = 21.80, *p* < 0.001 η*^2^* = 0.08; (b) Language of Intervention and PM point, *F*(2,228) = 3.09, *p* = 0.047, η*^2^* = 0.03; (c) Language of Intervention, Language of Testing, and PM point, *F*(2,228) = 4.64, *p* = 0.01 η*^2^* = 0.04; (d) Language of Intervention, Language of Testing, and Group (experimental/control) *F*(1,228) = 41.08, *p* < 0.001 η*^2^* = 0.15; and (e) Language of Intervention, Language of Testing, Group, and PM point, *F*(2,228) = 5.59, *p* = 0.004, η*^2^* = 0.05.

**FIGURE 2:**
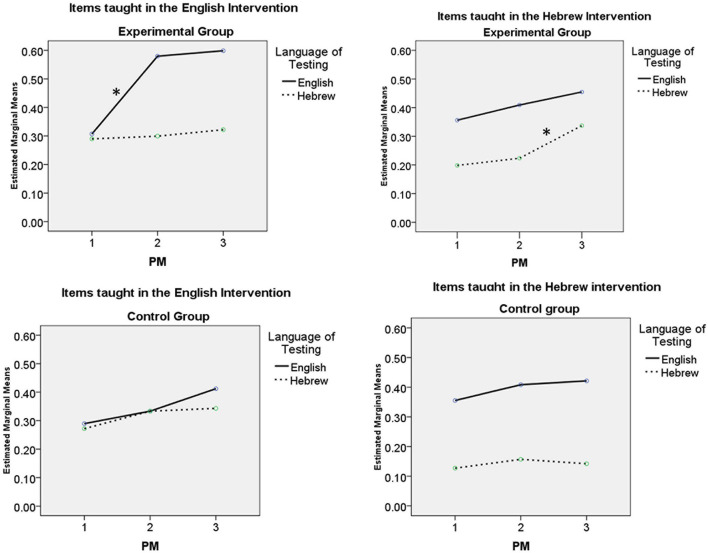
Growth of vocabulary knowledge in English and Hebrew.

Figure 2 provides a visual representation of the growth of vocabulary knowledge in each language (presented as ratios of correct responses) from one PM point to the next as a function of language of intervention. Figures 2A,B present growth in the ratio of vocabulary knowledge in English and Hebrew testing for items taught in English (2a) and for items taught in Hebrew (2b) for the experimental group. Figures 2C,D presents the same information for the control group.

Figure 2 and the GLM demonstrate that following intervention in HL/English, only children in the experimental group (Figure 2A vs. Figure 2C, English line from PM point 1 to 2) showed growth in vocabulary in HL/English for the target items presented in the intervention. Following intervention in SL/Hebrew (Figure 2B vs. Figure 2D, Hebrew line versus English line from PM point 2 to 3), children showed growth in SL/Hebrew vocabulary for those target items taught in the intervention, with no change in the translation equivalents that were not subject to intervention. Children in the control group showed no such effects (Figures 2C,D, English line from PM point 1 to 2, Hebrew line from PM point 2 to 3). That is, bilingual narrative intervention with vocabulary instruction had a direct impact on lexical breadth in both the HL and SL lexicons of English-Hebrew preschool children, for the target items for which intervention was provided.

Further insights into the impact of the intervention within each language emerge from the immediate gains of the experimental group in each language following each intervention block. As can be expected following the results above, following the intervention in English significantly larger gains are observed in English (*M* = 0.27) than in Hebrew (*M* = 0.05) for target words that were taught in English, *t*(21) = 5.93, *p* < 0.001, and following the intervention in Hebrew larger gains are observed in Hebrew (*M* = 0.11) than in English (*M* = 0.02) for target words that were taught in Hebrew, and the difference was nearly significant, *t*(21) = 2.04, *p* = 0.054.

### Impact of Intervention on Lexical Depth in the HL and SL Lexicons

To understand the impact of the intervention on lexical depth, the quality of the definitions was explored for each item separately, using a 0–1–2 scoring scheme. Descriptively, improvement for the experimental group was observed in English for all target items taught in English after the English intervention (at PM2), and in Hebrew for all target items taught in Hebrew after the Hebrew intervention (at PM3). A Kruskal Wallis Test showed significant improvement in HL/English in the definitions, as a measure of lexical depth, between the first PM point at baseline and the second PM point after the English intervention for seven of the twelve items presented in the English intervention: damp (*p* = 0.017), cooperate (*p* = 0.008), narrow (*p* = 0.001), wise (*p* = 0.008), hidden (*p* = 0.007), repair (*p* < 0.001), and tremble (*p* < 0.001). This was reflected by an increase in the number of complete answers. Such a significant improvement was observed in Hebrew following the Hebrew intervention for only one item (suffocate, *p* = 0.011). No differences were found for the control group for any item in any language.

In sum, intervention in English affected the breadth and depth of lexical knowledge in English, while intervention in Hebrew mostly influenced breadth in Hebrew with very limited effect on depth of lexical knowledge. No differences between the PMs were observed for the control group on any of the measures. Thus, all further analyses to test for learner characteristic modulators and CLI (RQ2) focus only on the experimental group.

### Learner Characteristics and Cross-Language Influences

Our quantitative results so far show an impact of bilingual intervention on lexical breadth and depth only for the language of intervention, with no evidence for CLI. Yet, the large variability in learner characteristics within the group might have impacted the ability of the children to draw maximal benefits from the intervention, transferring gains across languages. Learner characteristics include the bilingual experience (chronological age, age of onset of bilingualism and length of exposure) and proficiency in each language at the onset of the intervention (standardized tests, familiarity with the vocabulary introduced in the intervention at baseline).

To address the large variance in these variables and identify significant relationships between learner characteristics and gains, non-parametric correlational analyses (Spearman) were conducted for the gains between PM1 and PM2 and between PM2 and PM3 separately for each language of testing for target words that were taught in the particular language and their translation equivalents. Of special interest for CLI are the relations between learner characteristics and possible gains for translation equivalents (words that were taught in the other language), that is, gains in Hebrew between PM1 and PM2 for words taught in English, and gains in English between PM2 and PM3 for words taught in Hebrew. Due to the high correlation between length of exposure to the SL and AoB, only AoB was used in the correlations and the following regressions.

Table 5 presents the correlation matrix of individual differences in chronological age, AoB, Hebrew proficiency, English proficiency and English vocabulary dominance at PM1 against HL and SL gains, separating target words from translation equivalents within each language. The upper right triangle of the matrix presents correlations for English while testing in English and the lower left triangle (italicized) presents correlations for Hebrew while testing in Hebrew. Numbers at the top (1–9) correspond to the measures in the first column on the left, but in the language of testing (English). Target words are words taught in the language of testing and translation equivalents are words taught in the other language. Significant correlations are in bold print.

**TABLE 5:** Correlation matrix of individual difference with HL and SL gains for target items and translation equivalents for each language separately.

	**English testing**								
**Hebrew testing**	**1**	**2**	**3**	**4**	**5**	**6**	**7**	**8**	**9**
1. Age in months	_	0.396	0.016	0.051	0.356	*−*0.129	*−*0.152	*−*0.146	*−*0.060
2. AoB	*0.396*	*_*	*−*0.353	0.017	*−*0.065	**0.423^∗^**	*−*0.300	0.199	*−*0.169
3. Hebrew proficiency	*0.016*	*−0.353*	_	*−*0.114	*−*0.369	0.103	0.343	0.168	0.186
4. English proficiency	*0.051*	*0.017*	*−0.114*	*_*	0.127	**0.434^∗^**	*−*0.141	0.177	*−*0.109
5. English dominance at PM1	*0.356*	*−0.065*	*−0.369*	*0.127*	_	*−*0.136	*−*0.398	*−* **0.471^∗^**	0.009
6. Target PM1_PM2	*0.280*	*−0.157*	*0.231*	*−* ***0.423^∗^***	*0.168*	*_*	*−*0.244	0.330	*−*0.010
7. Target PM2_PM3	*0.318*	*0.127*	*0.210*	0.613**	*0.174*	*−0.238*	_	0.281	0.112
8. Translation equivalent PM1_PM2	*0.178*	*0.138*	*0.344*	*0.170*	*0.120*	*0.149*	0.445*	*_*	*−*0.083
9. Translation equivalent PM2_PM3	*−0.027*	*−0.016*	*0.039*	*−0.150*	*−0.120*	*−0.006*	*0.158*	*−0.118*	_

*p < 0.05 and **p = 0.002.

*The upper right triangle in the matrix presents correlations for English while testing in English and the lower left triangle (italicized) presents correlations for Hebrew while testing in Hebrew. Significant correlations are in bold print. Numbers at the top (1–9) correspond to the measures in the first column on the left, but in the language of testing (English). AoB, age of onset of bilingualism; Hebrew proficiency, raw Goralnik score; English proficiency, CELF CLS; English dominance, relative familiarity with the vocabulary at baseline; PM, progress monitoring; Target words, words taught in the language of testing; Translation equivalents, words taught in the other language.*

Of the learner characteristics, age of onset of bilingualism, English proficiency at the onset of the intervention, and English vocabulary dominance (higher scores in English than Hebrew at baseline), presented significant correlations with gains in English and Hebrew. Of particular interest for CLI is the correlation between gains in Hebrew for translation equivalents of English words at PM2 and gains in Hebrew on target words in PM3 (Spearman *Rho* = 0.445, *p* = 0.038). In an attempt to tease apart the relative contribution of each of these three predictors – AoB, English proficiency, and English vocabulary dominance – to the gains in English and Hebrew, linear regression analyses were conducted on the gains observed in the two languages with all predictors entered simultaneously. Three models came out significant.

The first model explores which learner characteristics predict direct gains from the English intervention. In the first regression, these three variables accounted for 36% of the variance in gains in English between PM1 and PM2 on target items that were taught in English, *F*(3,18) = 3.38, *p* = 0.04 (Model 1 in Table 6). Children with later AoB and those who started out with higher English proficiency gained more in English from the English intervention.

**TABLE 6:** AoB, English proficiency, and English vocabulary dominance as predictors in regression models.

**Predictor**	***B***	**Standard error**	**β**	***t***	***p***
**Model 1: Predictors of gains in English for English target words at PM2**					

AoB	0.005	0.002	0.423	2.244	0.038
English proficiency	0.007	0.003	0.440	2.280	0.035
English vocabulary dominance	−0.054	0.164	−0.063	−0.329	0.746
			*R*^2^ = 0.36 *F*(3,21) = 3.39, *p* = 0.04		

**Model 2: Predictors of gains in English for English translation equivalent (of Hebrew target words not taught yet) at PM2**					

AoB	0.002	0.002	0.198	1.135	0.271
English proficiency	0.006	0.003	0.366	2.048	0.055
English vocabulary dominance	−0.534	0.158	−0.606	−3.392	0.003
			*R*^2^ = 0.45 *F*(3,21) = 4.95, *p* = 0.01		

**Model 3: Predictors of gains in Hebrew for Hebrew target words at PM3**					

AoB	0.001	0.002	0.109	0.562	0.581
English proficiency	0.009	0.003	0.549	2.762	0.013
English vocabulary dominance	0.027	0.176	0.031	0.154	0.879
			*R*^2^ = 0.32 *F*(3,21) = 2.82, *p* = 0.07		

*AoB, age onset of bilingualism.*

The second model aims to find out if gains are extended beyond the taught items to the translation equivalents. In the second regression, the same three variables accounted for 45% of the variance in gains in English between PM1 and PM2 on items that were not taught yet (i.e., English translation equivalent of Hebrew target words taught after PM2 in the Hebrew intervention), *F*(3,18) = 4.96, *p* = 0.01 (see Model 2 in Table 6). Here, children who demonstrated Hebrew vocabulary dominance (i.e., they began with lower English vocabulary scores than Hebrew vocabulary scores at the onset of the intervention) gained more in English translation equivalents for items not taught yet. No predictors were found for gains in Hebrew between PM1 and PM2 that would count as predictors of CLI.

In the third model, the three variables accounted for 32% of the variance in gains in Hebrew between PM2 and PM3 on target items that were presented in the Hebrew intervention, but these gains were not significant *F*(3,18) = 2.82, *p* = 0.07 (see Model 3 in Table 6). Here children with better English proficiency gained from the intervention in Hebrew. No predictors were found for gains in Hebrew translation equivalents or in English between PM2 and PM3 that would count as predictors of CLI. That is, only gains in HL/English between PM1 and PM2 yielded significant models.

### Cross-Language Influence on Lexical Depth

Since the quantitative analyses did not show evidence of CLI, a qualitative analysis of the children’s responses was used to explore cross-language influence from the HL to the SL and from the SL to the HL for lexical depth. Two different types of indications of CLI were noted, one focused on the lexical item and one on the definition taught in the intervention. The first type was transfer of lexical information about a word in one language to a word in the other language. The second type was transfer of definitions taught in one language to the definitions in the other language. Both phenomena were documented in both directions, i.e., from HL to SL and from SL to HL.

An example of a target word that elicited both types of CLI (semantic and definitional) is the word “float.” In English, “float” includes the meaning of floating in the air or floating on the surface of water, but the Hebrew word “*tsaf*” can only mean floating on water. (To talk about floating in the air, one would use the word “*af”* = fly). The children were taught the word only in Hebrew, and the definition provided was “to stay on top of the water” (in Hebrew). When asked to define the Hebrew word “float,” six of the children initially overextended the definition of float from English and responded “fly” (which was scored as correct in English but incorrect in Hebrew). However, following the Hebrew intervention, none of the children responded “*af*” (fly) as an answer in Hebrew (though four still did so in English). References to water increased (in both languages, though more so in Hebrew than in English), and six of the children gave more precise definitions as a result of intervention in Hebrew (e.g., “to stay on top of the water,” instead of “going in the water”). This word shows that the children transferred concepts learned in one language to the other language, as well as definitions learned, even without any explicit instruction regarding the crosslinguistic connections.

There were other examples of the children possibly transferring concepts from one language to the other. The word “search,” taught in English, elicited Hebrew definitions of “look” or “see” for three children after the English intervention, which were close to the English definition of “look for.” Of note, “to look” in English was considered a partially correct definition, but in Hebrew “*l’histakel*” (“to look”) was scored as incorrect/less appropriate. One child, who when asked for a Hebrew definition of “*xazak*” (“powerful”), a word which had been taught in English, code-switched into English and said “powerful.” Generally, one would expect children of this age to use the more frequent, lexically unmarked (Raichlin et al., 2018) word “strong” in their codeswitching, so the choice of “powerful” seems to indicate a connection between the semantic representations of the words. Another example is “hidden,” taught in the English intervention. In Hebrew, the word “*nistar*” (hidden) has no phonological or morphological relation to “*l’haxbi*” (hide) as hidden and hide do in English. However, after being taught the word “hidden” in the English intervention, three children gave definitions during Hebrew testing that included the notion of hiding, which might indicate a connection between the English and Hebrew representations.

An indication of definitional CLI is exemplified in the word “cooperate” in English, defined in the intervention as “to work together.” Many of the children knew something about this word in Hebrew before the English intervention; they gave partially correct responses and associations in Hebrew such as “*shatef*” (to share), “*marshe*…” (to allow other kids to play with them), or “*leshatef et haxaverim*” (to include their friends). (This may be due to the fact that “share” and “cooperate” in Hebrew have a common root). However, following the intervention, 10 of the children improved their Hebrew definitions by giving responses in Hebrew that were similar to the English definition they were taught. Thus, the children improved their definitions in a given language by using the definitions taught in the other language, implying that CLI occurred.

## Discussion

The current study explored the effects of bilingual narrative intervention with embedded vocabulary instruction on lexical breadth and depth in English-Hebrew bilingual kindergarteners. Results showed that the intervention increased the breadth and depth of the experimental children’s vocabulary in their HL/English and lexical breadth in the SL/Hebrew. In terms of modulating factors, no effect was found for the modulating factors on CLI. Better HL performance was correlated with later AoB (later acquisition of SL) and higher HL language proficiency scores. The children with higher HL proficiency also responded better to the SL/Hebrew intervention, gaining more in lexical breadth than those with lower English proficiency. Children dominant in SL/Hebrew vocabulary at the outset of the study also gained more from the HL/English intervention. Finally, bidirectional CLI was found for semantic information in a qualitative analysis of children’s responses. Since no gains were detected in the control group, the following discussion focuses only on the experimental group, relating first to the findings for lexical breadth and depth, then to CLI, and finally to the influence of bilingual learner characteristics.

### Lexical Breadth and Depth

Gains following bilingual vocabulary intervention in this study are in line with previous research on vocabulary interventions, which show that they are effective for preschool children (Marulis and Neuman, 2010) and that bilingual interventions lead to progress in both languages (Restrepo et al., 2013). In the present intervention, the children gained relatively more in HL/English than in SL/Hebrew. For most of these children, SL/Hebrew was the weaker language as seen in their Hebrew proficiency scores, where nine of the children scored more than 1.25 SD below the mean. Future interventions should examine the effect of beginning the intervention in the school language, Hebrew, to compare the effects of beginning with the home vs. school language.

The findings for depth also differed for the two languages. For HL/English, the intervention led to an increase in the depth of the children’s knowledge while for SL/Hebrew the effect on depth was more limited. This can be attributed to the different proficiency levels of the children’s two languages. As explained by the Revised Hierarchical Model (Kroll and Stewart, 1994; Kroll and Ma, 2018), improved proficiency leads to more direct access to shared conceptual representations of words, with these stronger links resulting in enhanced performance on language tasks. This could explain the differential performance in the two languages of the children, with better performance in HL/English, the dominant language of this group.

### Crosslinguistic Influence

Analysis of semantic CLI across languages showed evidence for transfer of knowledge from the intervention, resulting in more precise definitions. We interpret this as a reflection of deeper lexical knowledge. This phenomenon was documented bidirectionally, both from HL to SL and from SL to HL. Semantic CLI finds support in the Revised Hierarchical Model (Kroll and Stewart, 1994; Kroll and Ma, 2018) which posits shared semantic representations between words and concepts and lexical connections between words, and activation of the lexicons of both languages. MacWhinney’s Unified Competition Model also explains this bidirectional CLI, as similar features in languages may influence each other. The findings of the present study provide details about how these connections and representations change as a result of bilingual narrative intervention with a focus on vocabulary. As reported above, the word “float,” which includes a broader semantic range in English (“float” can be used with air and water) than in Hebrew (“*tsaf*” = float can be used with water only) resulted in overgeneralizations in Hebrew due to CLI. Initially, children may assume semantic similarity or even one-to-one semantic mapping between translation equivalents until they are taught otherwise (Jarvis and Pavlenko, 2008). Indeed, following the intervention, where the children are given precise information, they improved and crosslinguistically transferred the lexical knowledge taught in the other language. The present study adds to others which show evidence for these connections (Singh, 2014; Floccia et al., 2020).

The results of the present study also provide support for the activation of both languages in bilinguals (e.g., Singh, 2014; Degani et al., 2018; Floccia et al., 2020). Once the child has heard a word in one language, both lexicons can be activated and the child can draw from the conceptual network, which contains words from both languages. As Mancilla-Martinez and Vagh (2013) note, information about concepts tied to vocabulary known in one language may facilitate acquisition of translation equivalents and transfer of linguistic information. Previous studies have reported crosslinguistic activation to be stronger from the dominant language to the non-dominant language (Singh, 2014), and bidirectional connections have been found as well (Floccia et al., 2020). In the current study, crosslinguistic associations were seen in both directions: from HL to SL and from SL to HL. Where CLI was found as a result of intervention, the shared mechanism underlying all language learning may have facilitated the CLI of lexical information (Cummins, 1981, 2008).

While gains for breadth did not show CLI from the language of intervention to the other language, children did produce codeswitching in their responses, which in the present context can be considered a form of CLI. Codeswitching was observed more in Hebrew than in English and more among the control group than the experimental group (see Table 3). These findings show that CLI was modulated by the intervention, which increased the children’s use of full or partial responses, making codeswitching redundant. CLI was also modulated by language dominance.

In spite of the evidence of transfer of lexical knowledge crosslinguistically, which deepened the children’s knowledge of words that had been partially acquired, we did not find evidence of increased lexical breadth in a given language as a result of intervention in the other language for the experimental group as a whole. However, the children who did show gains in Hebrew for translation equivalents following the English intervention (at PM2), showed further gains in Hebrew target words following the Hebrew intervention (at PM3). This suggests that the children who rely on CLI at PM2 benefit more from the intervention. That is, in the quantitative analysis of CLI, words taught in one language did not automatically lead to significant gains on those words in the other language for all children. It seems that the improvements in the children’s responses as a result of CLI occurred more in cases where the children knew something about a word, but learned more as a result of the intervention (improvement in depth), thus limiting the number of potential words influenced by instruction in the other language. Indeed, in some cases, it was possible for a child’s score to remain stable while still being affected by CLI, for example in cases where responses from earlier PMs received maximal scores for lexical breadth (which included partial or complete knowledge), but then improved even further after the intervention, although it was not reflected in an increase in the score.

Although some evidence has been found in previous studies for CLI involving lexical breadth (Armon-Lotem et al., 2020), many scholars have noted that lexical items are presumed to be learned one at a time in each language (David and Wei, 2008; Bialystok et al., 2010), and thus are unlikely to transfer automatically across languages without explicit connections being made, unless they are cognates (August et al., 2005). In the current study, if explicit connections had been made across languages [e.g., by providing translations of words learned, or by pointing out associations across languages, as suggested by Lugo-Neris et al. (2015)] it is possible that the children would have learned more words in the other language. It is also possible that additional follow-up questions for each word may have elicited more information about the children’s knowledge and would have revealed more evidence of growth as a result of CLI. A more differentiated scale may also have captured more growth in the children’s knowledge. Moreover, the scale was designed to measure semantic content, not definitional form. Since previous research has shown that formal definition is one aspect of lexical knowledge that transfers (Ordóñez et al., 2002; Pham et al., 2018), it is feasible that the design of the scale and the scoring did not allow all the transfer effects to be detected. Future research should address these issues.

### Bilingual Experience

Our quantitative results showed impact of bilingual intervention on lexical breadth and depth only for the language of intervention. A possible reason for this finding could have been variability within the group in their bilingual experience (chronological age, age of onset of bilingualism, and length of exposure) and variability in proficiency in each language at the onset of the intervention (standardized tests, familiarity with the 24 vocabulary items introduced in the intervention). These factors might have impacted the ability of the child to draw maximal benefits from the intervention.

The regression analyses showed that children with later AoB and/or higher English proficiency benefited more from the intervention in HL/English and showed greater improvement in lexical breadth (Model 1), which may be due to their larger HL lexicon. Moreover, following intervention in Hebrew (PM2 to PM3), these same children showed higher gains than those with lower English proficiency in English for English translation equivalents (model 2) and in Hebrew for the Hebrew target words taught in the intervention (Model 3). That is, the stronger one’s HL, the larger one’s direct (Models 1 and 3) and indirect (Model 2) gains from the intervention. This raises the question as to why. One answer may be related to the process by which words are acquired. At PM1 and PM2, the children are exposed to unfamiliar concepts they do not recognize but acquisition has begun. Once they build foundations, even in another language, their strong HL foundation allows them to make larger SL gains once intervention in the SL is internalized (Cummins, 1979, 1981; Lugo-Neris et al., 2015). Thus, the fact that the vocabulary intervention was provided first in the stronger language may have enhanced their word learning skills and provided a basis for learning in the weaker language.

Moreover, those children whose English vocabulary was smaller than their Hebrew vocabulary showed an increase in their English vocabulary breadth for the translation equivalents that were not taught yet (Model 2). Similarly, children with low proficiency in English demonstrated an increase for these same words in Hebrew before they were taught, showing that they might have improved their metalinguistic skills and better understood how to define words. That is, these children generalized the abilities from the items they learnt to those they did not learn in both languages. The gain in English can further be attributed to the fact that they had more room to grow since they started at a lower point. This finding is in contrast to the Matthew effect, where the rich get richer (Stanovich, 1986); those who are relatively “poorer” in the HL vocabulary gain more in the HL, suggesting that intervention focused on teaching vocabulary in a context-rich environment with multiple exposures to words and opportunities for practice may be a way of counteracting the Matthew effect and boosting the weaker language. However, since all of the children had the same order of intervention (HL first, SL second), the mechanism here is to be further researched. As mentioned above, future research manipulating the order of language of intervention will help to clarify this issue.

### Limitations

The population in this study consisted of typically developing children from mid-high SES levels with normal language proficiency and IQ scores and no comorbidities. It is therefore necessary in the future to examine whether these results are applicable to other demographics with various risk factors. Nevertheless, given the success of the intervention with this population, it is possible that this intervention program may have promise for helping atypical bilingual populations, such as children with developmental language disorders (DLD), to strengthen their language skills. Given the known difficulties of this population in expressive language, it is possible that a more structured task such as asking questions may be more fruitful for examining depth of knowledge. In addition, this study was focused on exploring CLI. Different words were intentionally taught in the two languages to test for CLI of conceptual knowledge. This specific design feature does not allow for a direct comparison between the items of the two interventions in the two languages, which is a potential limitation of the current study.

Another limitation relates to the data collection. Given that the onset of the Covid-19 pandemic coincided with a portion of the data collection, some of the data were collected online. Based on observation of the children in addition to their self-reports about their online experience as compared to the face-to-face sessions, it was concluded that the online mode did not alter children’s performance. In addition, the experience was generally positive and raises the possibility of continuing to use this method in the future. This result is in line with other studies that found children’s online and offline performance on language assessments to be comparable (Waite et al., 2010; Ciccia et al., 2011; Sutherland et al., 2017; Manning et al., 2020), but more research is needed to confirm these findings.

### Conclusion

The present study examines the effects of bilingual narrative-vocabulary intervention on lexical breadth and depth and evidence for CLI. The study shows that bilingual narrative intervention with vocabulary instruction may be efficacious for improving the lexical breadth and depth of bilingual kindergarten children, which may be critical for their future academic success (Dickinson et al., 2003; Han, 2012; Kieffer, 2012). Although no quantitative evidence for CLI was observed, this study provides additional evidence for the simultaneous activation of lexicons in both languages when using either language (Kroll and Stewart, 1994; Singh, 2014; De Anda and Friend, 2020), leading to CLI of lexical depth. This has implications for planning intervention, as it suggests that both languages may be used to facilitate lexical growth. In addition, as in previous research (Rolstad et al., 2005; Restrepo et al., 2013; Mendez et al., 2015), support of the HL did not hinder the development of the school language, and was efficacious in stimulating growth in school language knowledge, due to CLI. Later AoB (later acquisition of SL) and higher HL language proficiency were associated with better HL and SL performance, suggesting that a strong basis in the HL may enhance linguistic outcomes, providing further support for maintaining and strengthening the HL of bilingual children. Hence, this type of intervention may be used to support both languages of dual language learners, especially those whose HL is not taught in school, and who may be at risk for HL attrition (Ardila et al., 2019). The results here contribute to the body of research about the development of the lexical-semantic networks of bilingual children and highlight the importance of strengthening both the HL and SLs, as well as a method for doing so.

## Data Availability Statement

The raw data supporting the conclusions of this article will be made available by the authors, without undue reservation.

## Ethics Statement

The studies involving human participants were reviewed and approved by the Bar-Ilan University Institutional Review Board on Humanities and the Israeli Ministry of Education Chief Scientist. Written informed consent to participate in this study was provided by the participants’ legal guardian/next of kin.

## Author Contributions

ML, CA, JW, and SA-L were responsible for the conception, analysis, and interpretation of the work, and drafted and revised the article include intellectual content. CA was accountable for the integrity and accuracy of the work. SA-L and JW was approved the article for publication of content. All authors contributed to the article and approved the submitted version.

## Conflict of Interest

The authors declare that the research was conducted in the absence of any commercial or financial relationships that could be construed as a potential conflict of interest.

## Publisher’s Note

All claims expressed in this article are solely those of the authors and do not necessarily represent those of their affiliated organizations, or those of the publisher, the editors and the reviewers. Any product that may be evaluated in this article, or claim that may be made by its manufacturer, is not guaranteed or endorsed by the publisher.
