# Supplementary material for: Crosslinguistic Influence (CLI) of Lexical Breadth and Depth in the Vocabulary of Bilingual Kindergarten Children – A Bilingual Intervention Study
Source: Front Psychol. 2021 Sep 30;12:671928. doi: 10.3389/fpsyg.2021.671928 (PMC8516401; doi:10.3389/fpsyg.2021.671928)
Supplement: Supplementary file 1 [file Data_Sheet_1.pdf]

APPENDIX A: Intervention Session Protocol

During each intervention session, the children were exposed to the target words multiple times, in the context of a story and other contexts, and they used repetition, pictures, and acting out of the words to practice them. Each session lasted about 15-20 minutes.

These are the activities that made up each session:

1. **Vocabulary Review:** The vocabulary words learned in the previous session are briefly reviewed.

2. **Story Telling:** The experimenter reads a short story that includes two target vocabulary words (one verb, one adjective). The story is illustrated on six cards placed in front of the children, each one corresponding to a different part of the story.

3-4. **Introduce and Define Target Vocabulary Words One and Two:** Each word is introduced and defined using the context and pictures from the story. The experimenter and the children repeat the words and definitions several times (first one word, then the other).

5. **Rereading the Story (with Gestures Game):** The experimenter rereads the story, and the children make gestures they were taught indicating which part of the story is being read.

6. **Individual and/or Team Story Retelling:** Using the pictures, the children retell the story, either in groups (at earlier stages) or individually (at more advanced stages).

7-8. **Practicing Target Word One and Two:** The target words and definitions are repeated and practiced several times using a picture with a context that is different from the original story.

9-10. **Vocabulary Review Games:** The experimenter guides the children in short activities/games to practice the vocabulary words in other contexts, using repetition, pictures, and acting out the words.

APPENDIX B: Sentences for Vocabulary Knowledge Task

*Target Words are in Bold (E = English Target Word; H = Hebrew Target Word)

*The Hebrew sentences appear with transliteration and translations below them.

1. The girls **collected** rocks. (E)	1. הילדים **אספו** את המשחקים.<br>*ha-yeladim **asfu** et ha-misxakim.*<br>The children **collected** the toys.
2. The boxes are **heavy**. (H)	2. השקיות **כבדות.**<br>*ha-sakiot **kvedot**.*<br>The bags are **heavy**.
3. The woman is **powerful**. (E)	3. המורה **חזקה.**<br>*ha-mora **xazaka**.*<br>The teacher is **powerful**.
4. His hand is **sticking out**. (H)	4. הסלע ה**בולט** הוא אפור.<br>*ha-sela ha-**bolet** hu afor.*<br>The **sticking out** rock is gray.
5. They **searched** for their dog. (E)	5. הילד **חיפש** את המפתח.<br>*ha-yeled **xipes** et ha-mafteax.*<br>The boy **searched** for the key.
6. He looked at the **raised** sign. (H)	6. המורה הסתכלה על היד ה**מוגבהת.**<br>*ha-mora histakla al ha-yad ha-**mugbahat**.*<br>The teacher looked at the **raised** hand.
7. The boy **scrubbed** the floor. (E)	7. דני **שפשף** את האוטו.<br>*dani **shifshef** et ha-oto.*<br>Danny **scrubbed** the car.
8. The children are **leaping**. (H)	8. הארנבים **מנתרים.**<br>*ha-arnavim **menatrim**.*<br>The bunnies are **leaping**.
9. The children **cooperated**. (E)	9. הרקדנים **שתפו פעולה.**

	ha-rakdanim ***shitfu peula****.*<br>The dancers cooperated.
10. The woman **tripped**. (H)	10. מיכל **מעדה**.<br>*mixal* ***ma'ada****.*<br>Michal **tripped**.
11. She will **repair** the machine. (E)	11. הוא **תיקן** את המכנסיים.<br>*hu* ***tiken*** *et ha-mixnasayim.*<br>He **repaired** the pants.
12. The rock is **slippery**. (H)	12. הכביש **חלקלק**.<br>*ha-kvish* ***xalaklak****.*<br>The road is **slippery**.
13. The **damaged** refrigerator is at home. (E)	13. המחשב **הפגום** על השולחן.<br>*ha-maxshev ha-****pagum*** *al ha-shulxan.*<br>The damaged computer is on the table.
14. The ball **floated**. (H)	14. העלים **צפים**.<br>*ha-alim* ***tsafim****.*<br>The leaves are **floating**.
15. My hands are **trembling**. (E)	15. הילדה **רעדה**.<br>*ha-yalda* ***ra'ada****.*<br>The girl is **trembling**.
16. He **bumped into** a wall. (H)	16. הוא **נתקל** בעץ.<br>*hu* ***nitkal*** *ba-etz.*<br>He **bumped into** the tree.
17. The door is **narrow**. (E)	17. הרחוב **צר**.<br>*ha-rexov* ***tsar****.*<br>The street is **narrow**.
18. The garden is **spacious**. (H)	18. הארון **מרווח**.<br>*ha-aron* ***meruvax****.*<br>The closet is **spacious**.

19. The towel is **damp**. (E)	19. הכביסה **לחה**.<br>*ha-kvisa **laxa**.*<br>The laundry is **damp**.
20. The spider **escaped**. (H)	20. האיש **נמלט**.<br>*ha-ish **nimlat**.*<br>The man **escaped**.
21. The mother is **wise**. (E)	21. הילדה **נבונה**.<br>*ha-yalda **nevona**.*<br>The girl is **wise**.
22. The boy **crossed** the room. (H)	22. הילד **חצה** את הנהר.<br>*ha-yeled **xatsa** et ha-nahar.*<br>The boy **crossed** the river.
23. The **hidden** shoe was blue. (E)	23. התיק **המוסתר** אדום.<br>*ha-tik ha-**mustar** adom.*<br>The **hidden** bag is red.
24. It is **suffocating** in the house. (H)	24. היה **מחניק** באוטובוס.<br>*haya **maxnik** ba-otobus.*<br>It was **suffocating** on the bus.
